# Which criteria characterize a health literate health care organization? – a scoping review on organizational health literacy

**DOI:** 10.1186/s12913-021-06604-z

**Published:** 2021-07-06

**Authors:** Daniel Bremer, Izumi Klockmann, Leonie Jaß, Martin Härter, Olaf von dem Knesebeck, Daniel Lüdecke

**Affiliations:** 1 Department of Medical Psychology, Center for Health Care Research, University Medical Center Hamburg-Eppendorf, Martinistr. 52, 20246 Hamburg, Germany; 2 Department of Medical Sociology, Center for Health Care Research, University Medical Center Hamburg-Eppendorf, Martinistr. 52, 20246 Hamburg, Germany

**Keywords:** Organizational health literacy, Health literacy responsiveness, Health care organizations, Health literacy, Organizational development, Patient-centered communication, Scoping review

## Abstract

**Background:**

Organizational health literacy (OHL) aims to respond to the health literacy needs of patients by improving health information and services and making them easier to understand, access, and apply. This scoping review primarily maps criteria characterizing health literate health care organizations. Secondary outcomes are the concepts and terminologies underlying these criteria as well as instruments to measure them.

**Methods:**

The review was carried out following the JBI Manual on scoping reviews. The databases CINAHL, Cochrane Library, JSTOR, PsycINFO, PubMed, Web of Science Core Collection, and Wiley Online Library were searched in July 2020. Three researchers screened the records and extracted the data. The results were synthesized systematically and descriptively.

**Results:**

The literature search resulted in 639 records. After removing duplicates, screening by title and abstract, and assessing full-texts for eligibility, the scoping review included 60 publications. Criteria for OHL were extracted and assigned to six main categories (with 25 subcategories). The most prevalent topic of organizational health literacy refers to communication with service users. Exemplary criteria regarding this main category are the education and information of service users, work on easy-to-understand written materials as well as oral exchange, and verifying understanding. The six main categories were defined as 1) communication with service users; 2) easy access & navigation; 3) integration & prioritization of OHL; 4) assessments & organizational development; 5) engagement & support of service users, and 6) information & qualification of staff. The criteria were based on various concepts and terminologies. Terminologies were categorized into four conceptual clusters: 1) health literacy in various social contexts; 2) health literate health care organization; 3) organizational behavior, and 4) communication in health care. 17 different assessment tools and instruments were identified. Only some of the toolkits and instruments were validated or tested in feasibility studies.

**Conclusions:**

Organizational health literacy includes a significant number of distinct organizational criteria. The terminologies used in the OHL literature are heterogeneous based on a variety of concepts. A comprehensive, consensus-based conceptual framework on OHL is missing.

**Supplementary Information:**

The online version contains supplementary material available at 10.1186/s12913-021-06604-z.

## Background

Since the beginning of the 2000s, a new perspective on health literacy was introduced by focusing on the context, culture, and complexities of health care systems and health care organizations [1–3]. The idea of organizational health literacy (OHL) or health literacy responsiveness, defined as “the way in which services, organisations and systems make health information and resources available and accessible to people according to health literacy strengths and limitations” [4], was born. The concept arose from the acknowledgment that individual health literacy is considered an important issue for public health and health care [5]. Studies continue to show that large proportions of populations have insufficient or problematic health literacy [6, 7] and report even downward trends in health literacy over the last decade [8]. Limited health literacy is associated with a broad range of adverse health behaviors and health outcomes, e.g. causing poor self-efficacy regarding health-related issues, having less health-related knowledge, hindering medication adherence, generating higher rates of hospitalization, and provoking increased health care costs [9].

OHL focuses on the role of organizations to improve patient navigation through health care systems. It is consistent and important for health care organizations to respond to the health literacy needs of patients and to improve health information and services in order to make these more understandable and accessible [10–12]. Studies show that health care systems and their organizations do not react adequately to the existing and growing silent epidemic of insufficient health literacy [13–16]. Over the last 15 years, a considerable amount of research on OHL has been conducted to promote and evaluate changes in various organizational contexts, e.g. hospitals [17–21] or in primary health care settings [22–29].

Despite some specific literature reviews [20, 30–33], a consensus on criteria that characterize health literate health care organizations (HLHCOs) in different health care settings has not been reached. The reviews include some aspects of criteria that characterize HLHCOs, but they are limited to specific health care settings [20], focus on certain attributes of OHL [20, 30] and/or interventions [20, 33], their evaluation [31] and implementation [32]. Furthermore, comprehensive and current information on the criteria’s underlying concepts and terminologies, and on the instruments to measure these criteria, is expandable. Only a few studies investigated theories and concepts [30] and terminologies [34] of OHL. Both studies did not relate to the variety of criteria that characterize HLHCOs. The only review on instruments to measure OHL was performed in 2014 [35]. The study identified a large number of instruments to measure OHL, but focussed on only one database, certain attributes of HLHCOs [2], and quantitative measures.

This scoping review aims to first identify criteria that characterize HLHCOs across the literature. Secondly, it aims to describe concepts, terminologies, and instruments used to inform and measure these criteria. This review is part of the OHL-HAM study, a research project on OHL in the Hamburg Metropolitan Area (Germany) [36]. The aim of the study is to develop a consensus-based set of OHL criteria in consultation with various types of health care organizations.

## Materials and methods

Since our research questions aim at the identification of key characteristics, key concepts, and terminologies as well as measurement instruments related to OHL, a scoping review was conducted [37]. The scoping review followed the methodological framework of Arksey and O’Malley [38] and the guidelines of the JBI Manual for Evidence Synthesis [39, 40]. The reporting of this scoping review was based on the “Preferred Reporting Items for Systematic reviews and Meta-Analyses extension for Scoping Reviews (PRISMA-ScR) Checklist” [41] (additional file 1 “PRISMA-ScR Checklist”), the reporting guideline “Synthesis without meta-analysis (SWiM)” [42], and a study protocol including all preliminary specifications accessible on OSF Registries [43].

The study protocol included three further secondary research questions, which we also included in our data extraction and analysis. Due to the large amount of materials, we decided to omit detailed results concerning these three additional questions to avoid overburdening this manuscript. Instead, we included the respective results in the additional files section (additional file 2 “Process stages of becoming an HLHCO”, 3 “Involved actors by type of organization”, and 4 “Implemented interventions”) and refer to them in the discussion.

### Data sources and search strategy

After developing the research questions and performing a pilot run of literature search for configuring the search string, seven databases were used to gather the final records for our scoping review (3 July 2020). The final search was performed with CINAHL, Cochrane Library, JSTOR, PsycINFO, PubMed, Web of Science Core Collection, and Wiley Online Library. Using truncations and Boolean operators, the databases were searched for three sets of keywords referring to “organization*”, “health literacy/literate”, and “criteria/criterion/attribut*/policy/policies/guideline*/recommendation*” (additional file 5 “Search details”). Our search string followed the search strategy PICo (Population, Interest, Context) [44]. Search string and settings were adapted to the different databases.

### Eligibility criteria

Literature was included that covered the context of health care provision (hospitals, health centers, physicians, etc.), and healthcare-related organizations (e.g. patient organizations, health insurances, physicians and psychotherapist associations, etc.) in any health care setting. Publications were included if they addressed OHL criteria. Literature reviews were used to identify additional records during the snowball process but were not extracted for data synthesis. No restrictions were defined regarding the source of information to prevent excluding specific types of evidence. No geographic or temporal restrictions were defined. Records published in languages other than German or English were excluded.

### Screening process

A three-stage screening process was conducted to assess the relevance of the records. 1) Title and abstract screening: After merging the identified titles in a Citavi library (version 6), duplicates were removed and accessible abstracts were collected. Titles and abstracts of the remaining 301 records were screened by two pairs of researchers (DB, IK (n records = 120), and DL, IK (n records = 181)). The respective third researcher (DB/DL) was consulted for screening results deviating between the two initial researchers. Prior to the screening, two pilot screening phases, each covering 10% (randomly selected) of the records, were conducted by two researchers (DB, IK). 2) Full-text screening: After gathering all accessible full-texts, two researchers (DL, IK) conducted two pilot screening phases, each covering 10% (randomly selected) of the records. Subsequently, the two pairs of researchers conducted the screening of the full-texts, again consulting the respective third researcher (DB/DL) in case of deviations. 3) Snowballing: We examined the reference lists of the identified full-text publications in order to identify other relevant sources of information.

### Data extraction

The authors developed a standardized data extraction form, tailored to this scoping review. Before data charting, two reviewers (DB, IK) tested and adapted the extraction form to make sure that all relevant results were captured [38, 45, 46]. Data extraction and synthesis for the primary research question were done by one researcher (IK) and controlled by two researchers (DB, LJ). Data extraction for the other variables was done by three researchers (DB, IK, LJ). In case of uncertainty, the research team was consulted. A formal risk of bias or quality assessment was neither necessary nor possible due to the character of a scoping review [37].

Using the standardized form, we collected information on 1) author, 2) year, 3) country, 4) type of publication, 5) type of study, and 6) group of participants, 7) criteria of OHL, 8) terminologies of OHL, 9) conceptual background of the terminologies,^1^ 10) assessment tools & instruments, 11) methods of assessment, 12) processes to become an HLHCO, 13) type of organization, 14) actors, 15) service user, and 16) interventions to promote OHL.

To answer the primary research question, the records were screened for OHL criteria and related terms (e.g. attributes, health literacy responsiveness). The majority of publications structured the criteria into main (e.g. “communication”) and subcategories (e.g. “communicates clearly”). In the case of multilevel lists of criteria, we only extracted the lowest list level available. This made the inductive process of clustering categories more independent from the original main categories, which were not extracted in such cases. When different publications referred to the same criteria, the criteria were only extracted once. For example, a group of publications [18, 47–52] referred to the “Ten Attributes of Health Literate Health Care Organizations” [2].

### Synthesis of results

Our findings were synthesized descriptively and narratively to provide a systematic classification of OHL criteria as well as its underlying concepts and available instruments. Furthermore, we provide tables including frequency counts wherever possible. The extracted criteria were inductively clustered into newly developed main categories and subcategories. The included records were screened for concepts and terminologies of OHL to summarize how OHL in the context of OHL criteria is understood. The extracted terminologies were assigned to conceptual clusters. The publications using OHL assessment instruments were grouped by instrument.

## Results

### Search and screening process

**Fig. 1:**
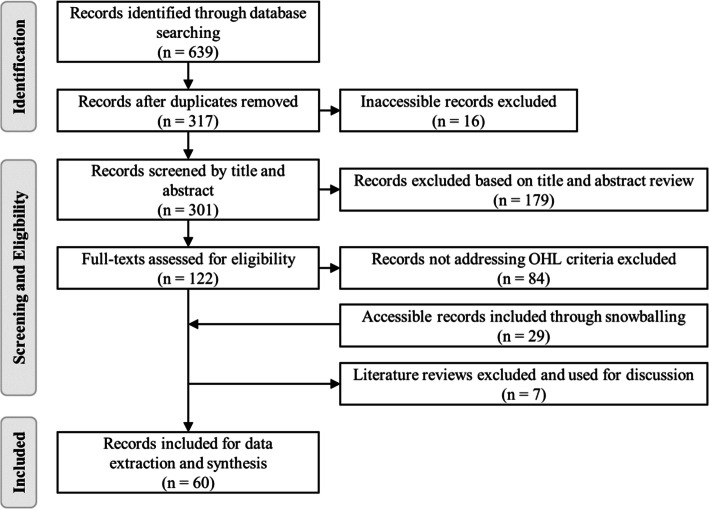
Flowchart of the screening process [53]

A total of 639 publications were identified through a database search. After removing 322 duplicates and 16 inaccessible records, 301 articles remained for title and abstract screening during which further 179 records were excluded. After screening the remaining 122 full-texts for eligibility, 84 more records were excluded. By screening the references of the records, 29 records were added and seven literature reviews were excluded (additional file 6 “Included and excluded records”). Finally, 60 publications were included for data extraction and synthesis (Fig. 1).

### Sample of included records

All 60 records included were published between 2006 and 2020 (Table 1).

**Table 1:** Characteristics of included records

Author, year (sorted), country	Type of publication (type of study)	Group of participants
Rudd & Anderson, 2006, USA [1]	Guidebook (Toolkit)	n. a.
Jacobson et al., 2007, USA [54]	Guidebook (Toolkit)	n. a.
NALA, 2009, Ireland [55]	Guidebook (Toolkit)	n. a.
DeWalt et al., 2010, USA [24]	Guidebook (Toolkit)	n. a.
Rudd, 2010, USA [56]	Guidebook (Toolkit)	n. a.
DeWalt et al., 2011, USA [26]	Journal article (Toolkit, feasibility std.)	Staff members (eight practices)
Brach et al., 2012, USA [2]	Journal article (Discussion)	n. a.
Institute of Medicine, 2012, USA [57]	Chapter/book (WS)	Representatives from HCO
Parker & Hernandez, 2012, USA [58]	Journal article (WS)	Representatives from HCO
Six-Means et al., 2012, USA [29]	Journal article (Implementation, case std.)	n. a.
Weaver et al., 2012, USA [59]	Journal article (Case std.)	19 staff members, 16 patients (one health center, three clinics)
Wong, 2012, USA [52]	Journal article (Discussion)	n. a.
Institute of Medicine, 2013, USA [60]	Chapter/book (WS)	Health literacy implementers from HCOs
O’Neal et al., 2013, USA [61]	Journal article (Assessment, feasibility std.)	45 staff members, 62 patients (eight pharmacies)
Thomacos & Zazryn, 2013, Australia [49]	Guidebook (Toolkit)	n. a.
Abrams et al., 2014, USA [62]	Guidebook (Toolkit)	n. a.
Brach et al., 2014, USA [23]	Journal article (Discussion)	n. a.
Johnson, 2014, Australia [63]	Journal article (Case std.)	Two consumers (one HCO)
Kaphingst et al., 2014, USA [64]	Journal article (Cross-sectional std.)	3358 adults (general population)
Palumbo & Annarumma, 2014, Italy [65]	Journal article (Evaluation std.)	40 healthcare professionals (various HCOs)
Schuman, 2014, USA [22]	Chapter/book (Discussion)	n. a.
Altin & Stock, 2015, Germany [66]	Journal article (Discussion)	n. a.
Altin et al., 2015, Germany [25]	Journal article (Validation std.)	1125 adults (general population)
Brega et al., 2015, USA [67]	Guidebook (Toolkit)	n. a.
Briglia et al., 2015, USA [68]	Journal article (Case std.)	One health care network (12 health centers)
Kowalski et al., 2015, Germany [19]	Journal article (Validation std.)	51 directors, 1224 patients (56 hospitals)
Ministry of Health, 2015, New Zealand [69]	Guidebook (Toolkit)	n. a.
Pelikan & Dietscher, 2015, Austria [70]	Guidebook (Toolkit, feasibility std.)	Three to eleven staff members per hospital (nine hospitals)
Pelikan & Dietscher, 2015, Austria [71]	Journal article (Toolkit, feasibility std.)	n. a.
Annarumma & Palumbo, 2016, Italy [72]	Journal article (Evaluation std.)	40 healthcare professionals (various HCOs)
Dietscher & Pelikan, 2016, Austria [17]	Journal article (Toolkit, feasibility std.)	Three to 11 staff members per hospital (nine hospitals)
Innis, 2016, Canada [73]	Doctor thesis (Pilot std.)	42 health care experts; 99 staff members (79 hospitals)
Napel, 2016, USA [74]	Doctor thesis (Cross-sectional std.)	102 nurses (various hospitals)
Adsul et al., 2017, USA [75]	Journal article (Qualitative std.)	11 senior staff members (11 HCOs)
Baur et al., 2017, USA [76]	Chapter/book (Discussion)	n. a.
Brach, 2017, USA [10]	Journal article (Discussion)	n. a.
Dietscher & Pelikan, 2017, Austria [77]	Chapter/book (Toolkit, feasibility std.)	Three to 11 staff members per hospital (nine hospitals)
Eigelbach, 2017, USA [78]	Journal article (Discussion)	n. a.
Ernstmann et al., 2017, Germany [79]	Journal article (Validation std.)	Five experts; 453 patients (18 cancer centers)
Farmanova, 2017, Canada [80]	Doctor thesis (Qualitative std.)	12 health service org.; nine managers (eight health service org.)
Palumbo et al., 2017, Italy [81]	Journal article (Multiple case std.)	15 experts (three hospitals)
Prince, 2017, USA [47]	Doctor thesis (Case std.)	463 employees (one health center)
Trezona et al., 2017, Australia [12]	Journal article (Assessment, evaluation std.)	42 professionals; 153 professionals (36 health and social service org.)
Wieczorek et al., 2017, Austria [82]	Journal article (Assessment, evaluation std.)	Six to 15 youth workers/federal state (six youth work settings in three fed. states)
Hayran & Özer, 2018, Turkey [83]	Journal article (Cross-sectional std.)	18 managers and 1459 patients (three hospitals)
Leonard et al., 2018, USA [84]	Journal article (Case std.)	One medical center
Oelschlegel et al., 2018, USA [85]	Journal article (Case std.)	One medical center
Palumbo & Annarumma, 2018, Italy [86]	Journal article (Assessment std.)	16 senior managers/staff members (16 pharmacies)
Prince et al., 2018, USA [18]	Journal article (Case std.)	463 employees (one health center)
Trezona et al., 2018, Australia [87]	Journal article (Toolkit)	22 health/social service professionals (22 org.)
Trueheart, 2018, USA [50]	Doctor thesis (Multiple case std.)	13 experts (13 health org.)
Brega et al., 2019, USA [88]	Journal article (Delphi std.)	18 experts (20 org.); one patient representative
Kaper et al., 2019, Ireland, Netherlands [89]	Journal article (Longitudinal mixed-methods std.)	24 staff members, 40 service users (four hospitals)
Pelikan, 2019, Austria [90]	Chapter/book (Discussion)	n. a.
Vamos et al., 2019, USA [51]	Journal article (Development of teaching tool)	One pediatric health clinic
Aaby et al., 2020, Denmark [91]	Journal article (Case std., feasibility std.)	Three managers and four therapeutic teams (one rehabilitation unit)
Bonaccorsi et al., 2020, Italy [92]	Journal article (Cross-sectional std.)	405 healthcare managers (20 hospitals)
Goldsmith et al., 2020, USA [93]	Journal article (Framework)	n. a.
NASEM, 2020, USA [94]	Chapter/book (WS)	Patients; health care professionals; various stakeholders (various org.)
Rathmann et al., 2020, Germany [48]	Journal article (Explorative std.)	130 managers/staff (130 facilities for people with disabilities); eight staff members (eight facilities for people with disabilities)

*n* = 60 records; abbreviations: org. = organization(s), std. = study, WS = workshop summaries

Until 2013, most of the records were published in the USA, and in total, more than half of the records (n = 33) were published there. The remaining publications were conducted in six European countries (Austria, Denmark, Germany, Ireland, the Netherlands, Italy; n = 19), Australia (n = 4), Canada (n = 2), New Zealand (n = 1), and Turkey (n = 1).The included publications were grouped into four types: guidebook (n = 10), journal article (n = 38), chapter/book (n = 7), and doctoral thesis (n = 5). The type of study referred to the wording used in the publications or was assigned by the authors of this review. The majority was classified as toolkits (n = 15), discussions (n = 9), case studies (n = 9), feasibility studies (n = 7), cross-sectional studies (n = 4), evaluation studies (n = 4) or workshop summaries (n = 4).

### Synthesis

#### Criteria characterizing HLHCOs

In total, 490 criteria were extracted from 60 publications (Table 2 and additional file 7 “OHL criteria by category”). In total, four criteria were assigned to two different clusters resulting in the assignment of 494 criteria including duplicates.

**Table 2:** Criteria and attributes of HLHCOs

Main categories and subcategories	Number of extracted criteria
**1. Communication with service users**	**190**
1.1 Education & information	60
1.2 Easy to understand written materials & oral exchange	45
1.3 Verification of understanding & exchange	29
1.4 HL principles of communication	16
1.5 Diversity & awareness	15
1.6 Soft skills	13
1.7 Technology & innovation	12
**2. Easy access & navigation**	**86**
2.1 Navigating health care services & cooperation	37
2.2 Physical access & navigation	24
2.3 Provision of information & staff assistance	10
2.4 Telephone & online navigation	8
2.5 Overall ease	7
**3. Integration & prioritization of OHL**	**67**
3.1 Commitment, integration into planning	35
3.2 Dedication of resources	21
3.3 Dissemination of OHL	8
3.4 External factors	3
**4. Assessment & organizational development**	**57**
4.1 Evaluation, assessment, research, quality management	46
4.2 Needs identification	7
4.3 Transformation & development	4
**5. Engagement & support of service users**	**55**
5.1 Consultation & engagement of service users	30
5.2 Support for self-management	18
5.3 Family & caregivers	7
**6. Information & qualification of staff**	**39**
6.1 Organizational and individual health literacy of staff	19
6.2 Communication techniques	16
6.3 Professional development	4
**Total number of criteria** (including four duplicates)	**494**

60 records screened [1, 2, 10, 12, 17–19, 22–26, 29, 47–52, 54–94]; 30 unique sets of criteria [1, 2, 12, 17, 19, 24, 29, 30, 48, 49, 55–57, 59, 62, 65, 67, 69–71, 76, 77, 87, 88, 93–98]

By looking at each criterion separately, the following six main categories of criteria were identified: 1) communication with service users, 2) easy access & navigation, 3) integration & prioritization of OHL, 4) assessments & organizational development, 5) engagement & support of service users, and 6) information & qualification of staff. The six main categories were refined into 25 subcategories (Table 2). The number of criteria per category (last column in Table 2) shows how often they were addressed in the included publications. The frequency of a category does not necessarily equate to its importance but can illustrate an initial relationship between the criteria characterizing an HLHCO.

The most prevalent main category refers to the *communication with service users*, for example, patients, clients, family, or caregivers (n criteria = 190). Subcategories refer to a range of aspects such as educating and informing people by “provid[ing] patient training and assistance around personal health records and health IT tools” [57] or supporting communication by “set[ting] up systems that make checking for understanding standard practice” [62]. An important aspect of educating people and health promotion is that the organization aims to reach people beyond its patients in treatment and strives to “improve HL [health literacy] in the organisation’s community and catchment area” [98]. The second most represented main category is *easy access & navigation* (n criteria = 86). Criteria of this cluster, for example, refer to physical access & navigation meaning that “the organization has an easy-to-follow navigation system and signage” [77]. Navigating the health care system involves different health care services, requiring coordination and cooperation among the health care organizations. Hence, one aim for HLHCOs should be to “make referrals easy” [67].

The main category *integration & prioritization of OHL* (n criteria = 67) refers to the commitment of an organization, e.g. by integrating OHL into its planning and dedicating resources. An HLHCO “has leadership that makes health literacy integral to its mission, structure, and operations” [2]. External factors framing the implementation of criteria can be found in the “external policy and funding environment” [87]. HLHCOs monitor and improve their work with *assessments & organizational development* (n criteria = 57). They “use assessments to determine their performance and progress in promoting health literacy” [49]. The fifth main category refers to the *engagement & support of service users* (n criteria = 55). For example, navigation systems, services, and materials are designed by involving the peoples’ perspective, “undertaking community consultation and enabling consumer participation” [87]. Finally, for implementing all these criteria the *information & qualification of staff* (n criteria = 39) is pertinent for an HLHCO. Communication techniques are improved by “staff skills building (print communication and oral exchange)” [1] and “health promotion for staff” [48] is provided. Additional file 7 (“OHL criteria by category”) shows based on which criteria the categories were formed.

#### Underlying concepts and terminologies of OHL criteria

The authors defined four conceptual clusters (Table 3). The first cluster, “health literacy in various social contexts” (n = 18 records), considered the macro level and includes several societal areas, e.g. health care, education, cultural and social aspects linking them to health literacy and a system’s perspective (e.g. health literacy framework). The second cluster was defined by the term “health literate health care organization” (n = 70 records) (e.g. health literate hospitals and health centers). Organizational behavior (n = 9 records) was the third cluster, which pooled concepts of organizational behaviors and their procedural character (e.g. organizational responsiveness, organizational learning, or transformational change). Finally, the fourth cluster “communication in health care” (n = 6 records) focused on the micro-level pointing out various aspects of health literacy and communication in health care (e.g. communication quality, cultural factors, or patient-centered communication).

**Table 3:** Concepts and terminologies in publications on HLHCOs

Concept cluster Extracted terminology	Introducing reference	Referring references	n*
**1. Health literacy in various social contexts**			**18**
Health literate care	[99]	[64, 66, 73, 84]	4
Health literacy framework	[3]	[1, 26, 52, 54, 56, 61, 63, 65, 66, 72, 80]	11
	[100]	[70, 77, 82]	3
**2. Health literate health care organization**			**70**
Health literate organization (referring to health care)	[33]	[89]	1
Organizational health literacy (referring to health care)	[30]	[88, 89]	2
Health literacy environment of hospitals and health centers; literacy-friendly healthcare facility	[1]	[2, 24, 26, 52, 54, 55, 63, 65, 66, 69, 84]	11
Literacy friendly healthcare settings	[55]	[55]	1
Contextualizing health literacy to health care organizations	[72]	[89]	1
Health literate hospitals and healthcare organizations; Organizational health literacy of hospitals; Concept of the health literate health care organization	[71, 72, 77, 98]	[71, 77, 89, 90, 98]	2
Health literate health care organization	[2]	[10, 12, 17–19, 22, 23, 25, 29, 47–52, 58–60, 62–66, 68–80, 82–90, 92, 93]	47
	[57]	[49, 57, 58, 80, 81]	5
**3. Organizational behavior**			**9**
Health literacy universal precautions	[24]	[22, 52, 62, 88]	4
Organizational capacity for health literacy	[101]	[81]	1
Organizational health literacy responsiveness	[12, 87]	[89, 91]	2
Organizational learning	[102]	[73]	1
Organizational model for transformational change in health care systems	[97]	[75]	1
**4. Communication in health care**			**6**
Communicating health	[103]	[1]	1
Health literacy and communication quality in health care organizations	[104]	[72]	1
Literacy, culture, language to improve health care quality	[105]	[2]	1
Patient navigation support for health literacy and patient-clinician communication	[106]	[94]	1
Cultural framework for health	[107]	[94]	1
Patient-centered communication	[108]	[94]	1

[98] cited after [90, 97] cited after [75]; 60 records screened and extracted from; multiple assignments allowed; *n = number of records

In terms of concepts used, the majority of the included records (47 out of 60) referred to the publication “Ten Attributes of Health Literate Health Care Organizations” by Brach et al. [2]. Accordingly, an HLHCO is defined as “an organisation that makes it easier for people to navigate, understand, and use information and services to take care of their health” [2]. 11 records (see Table 3 “Health literacy framework”) related to the work of the Institute of Medicine by stating that “health literacy goes beyond the individual obtaining information. Health literacy emerges when the expectations, preferences, and skills of individuals seeking health information and services meet the expectations, preferences, and skills of those providing information and services. Health literacy arises from a convergence of education, health services, and social and cultural factors” [3]. The publication “The Health Literacy Environment of Hospitals and Health Centers. Partners for Action: Making Your Healthcare Facility Literacy-Friendly” by Rudd & Anderson [1] was the conceptual basis of 11 records. The remaining publications were extracted five times or less.

#### Instruments to measure OHL criteria

In total, 17 assessment tools and instruments were extracted from 36 records (Table 4). The “Health Literate Health Care Organization 10 Item Questionnaire (HLHO-10)” [19], “Health Literacy Universal Precautions Toolkit” [24, 67], and the “Health Literacy Environment of Hospitals and Health Centers (HLEHHC)” [1] were referenced the most within our sample of 60 records. Only some of the toolkits and instruments were validated or tested in feasibility studies [25, 26, 61, 70, 79, 91]. The methods of assessment were multifaceted, e.g. standardized questionnaires, semi-structured interviews, observations, checklists, and material assessments. Mainly, the existing tools were applied as surveys via questionnaires. The tools were either used in their original form (e.g. [19]) or by selecting a subset of items or tools (e.g. [86] using the Pharmacy Health Literacy Assessment Tool [54]), translating the tool into a different language [83], extending and adapting the contents [73] or adapting the measures for a different type of method [64].

**Table 4:** Instruments to measure the health literacy of health care organizations

Assessment tools & instruments	Applied by records
1. Communication Climate Assessment Toolkit (C-CAT) [104]	[65, 72]
2. Consumer Assessment of Health Providers and Systems (CAHPS® Clinician & Group Survey) [109]	[64]
3. Enliven Organisational Health Literacy Self-assessment [49]	[49]
4. Health Literacy Environment of Hospitals and Health Centers (HLEHHC) [1]	[1, 59, 81, 84, 85]
5. Health Literacy Review [69]	[69]
6. Health Literacy Universal Precautions Toolkit [24, 67]	[24, 26, 59, 67, 74, 80]
7. Health Literacy-Sensitivity of Communication (HL-COM) [79]	[79]
8. Health Literate Discharge Practices [96]	[73]
9. Health Literate Health Care Organization 10 Item Questionnaire (HLHO-10) [19]	[18, 19, 47, 48, 80, 83, 92]
10. Health Literate Primary Care Practice Screener (HLPC) [25]	[25]
11. Literacy Audit for Health Care Settings [55]	[55, 89]
12. Organisational Health Literacy Responsiveness (Org-HLR) self-assessment tool and process [87]	[87, 91]
13. Patient Education Materials Assessment Tool (PEMAT) [110]	[84]
14. Pharmacy Health Literacy Assessment Tool [54]	[54, 61, 86]
15. Quickscan Health Literacy Toolbox [111]	[89]
16. The Health Literacy Environment Activity Packet [56]	[56, 63]
17. Vienna Concept of Health-Literate Hospitals and Healthcare Organizations (V-HLO-I) [70]	[17, 70, 77]

Based on the screening of 60 records and data extraction from 36 records

## Discussion

### Summary of findings

This scoping review provides a comprehensive overview of OHL of health care organizations by synthesizing the current status of publications on criteria that characterize HLHCOs. Furthermore, we gathered information on which understanding of OHL these criteria are based on and how they are measured.

Our review included 60 publications on OHL criteria. We extracted 490 criteria (494 with duplicates) characterizing HLHCOs which were clustered into six main categories (Table 2). Rowlands et al. [31] stated a similar number and nature of categories in a literature review including some frameworks. In their recent review, Zanobini et al. [20] focused on hospitals, their characteristics, and interventions regarding OHL, and found that most of the included studies focused on contents easy to understand.

In line with this assessment, the largest main category of criteria identified by our review was *communication with service users*. More than half of the criteria assigned to this category were clustered into *education & information* and *easy to understand written materials & oral exchange*. While materials for patients are one of the main areas of work for OHL, we found a variety of other aspects relevant to the implementation of OHL criteria. Next to physical access to health care facilities, examples range from the participation and commitment of consumers to the organization’s commitment to OHL to a degree that OHL is widely integrated into its planning. An organization’s efforts to advocate for health literacy go beyond treating and educating its users. Instead, an HLHCO is also committed to promoting the health of its staff [2, 17, 48, 77, 95, 98] and the regional community [17, 76, 77, 98].

By investigating OHL guides, Farmanova et al. [30] showed that all reviewed guides included the items *communication* and *access and navigation* and that the *workforce* plays an important role not only in health care but also in the creation of a health literate environment. Furthermore, *leadership* was crucial by integrating health literacy in an organization’s vision, mission, and strategic planning [30].

Our review underlines the large number of OHL-related criteria indicating that OHL is a complex construct potentially including a significant number of organizational criteria that specify and expand the “Ten attributes of health literate health care organizations” [2, 12, 67, 90]. Consequently, becoming a health literate health care organization requires a holistic approach [90].

In our review, 60 records were screened for concepts and terminologies related to health literacy and organizational contexts (Table 3). The terminologies were extracted and assigned to four conceptual clusters. The concepts of Brach et al. [2], Institute of Medicine [3], and Rudd & Anderson [1] were the dominating ones in this context.

Meggetto et al. [34] identified 19 different terms to describe OHL, the three most frequent terms being *health system health literacy*, *organizational health literacy*, and *health literacy practice/s*. They observed that different terms overlap in content, but aim at different levels or contexts and concluded an interdependence between the terms and concepts: “Within health system health literacy sits OHL, and within this sits health literacy practice” [34].

Our results substantiated that a broad range of OHL terminologies based on various concepts exist in the OHL literature. This versatility represents the multi-level approach that is needed to incorporate the concept of OHL. An integrative framework of OHL could include micro- (e.g. individual behavior), meso- (e.g. organizational processes), and macro-level (e.g. health policies) aspects [90].

Our review shows that 17 different assessment tools and instruments were used (Table 4). The methods of assessment were versatile, but most of them used surveys via questionnaires. In their review, Kripalani et al. systematically described existing measures and found that “most measures have been developed with strong content validity, but little research has been done to examine their internal reliability, construct, or predictive validity” [35]. Rowlands et al. also “found limited evidence of the use of organizational health literacy/responsiveness measures and tools as part of an evaluation of a programme or intervention” [31]. Lloyd et al. stated in their study, searching for effective strategies for creating OHL, that “the use of health literacy tools proved important for raising awareness of health literacy issues within organisations, these tools were insufficient for generating the organisational changes necessary to improve organisational health literacy” [33].

Our review confirmed that a large number of toolkits and instruments to measure and assess OHL was published. Most of them were developed inductively, but with a practical orientation by including health care experts and service users [12, 87]. Only a few of them were validated or showed proof of feasibility [19, 77, 91].

To prevent overloading this manuscript, not all results are shown in detail. Following our study protocol, we also analyzed data on further involved persons and stakeholders (additional file 3), organizational processes (additional file 2) and implemented interventions to become an HLHCO (additional file 4). Our analyses showed that the involvement of service users either as part of a (potential) target group for an OHL instrument or in an active role during the implementation process itself still happens too rarely. More interventions and evaluations regarding activities of organizational change on OHL, such as the preparation and assessment stage of processes, are needed. Health service research should be included and consulted to support and monitor processes to become an HLHCO.

### Limitations

Literature reviews have to deal with the risk of publication bias. We were able to minimize it by using seven databases, snowballing techniques, and no restrictions regarding the source of information. The scoping review captured different types of publications (e.g. guidebooks, journal articles, doctoral thesis, and book chapters) to give a comprehensive overview of the subject. Nevertheless, some relevant records may have been omitted due to the absence of pertinent keywords in the search strings. Due to the characteristics of a scoping review, the quality of the publications was not evaluated. Notably, some of the presented toolkits and instruments were validated or tested in feasibility studies [25, 26, 61, 70, 79, 91]. Furthermore, the findings may be limited to health care organizations in Western countries, since more than two-thirds of the records (42 out of 60) were from North America (USA = 33, Canada = 2) and other English-speaking countries (Australia = 4, Ireland = 2, New Zealand = 1). Lastly, only English- and German-language publications were taken into account due to time and cost considerations.

## Conclusions

The idea of OHL turned the tables. It is not the patients and other service users who need to adapt, it is the health care organization’s responsibility to make “it easy for people to navigate, understand, and use information and services to take care of their health” [10]. This study showed which criteria of health care organizations make an organization health literate, which underlying concepts or terminologies exist, and what instruments are used to measure OHL criteria.

Since OHL is a complex construct, future research should focus on providing evidence on effective and efficient core elements of OHL to support the implementation of OHL criteria into health care practice. Furthermore, a consensus-based framework of organizational health literacy that is theoretically sound is still needed. Health services research should provide validated tools and instruments which are sensitive, but also feasible and easy for health care organizations to integrate into their daily routine. Before starting their “journey to become a health literate organization” [10] health care organizations should carefully define theirs aims, assess theirs needs and plan their resources. Considering that, the large number of OHL-related criteria may imply the necessity to focus on just a few aspects of OHL in the beginning.

## Supplementary Information

**Additional file 1.** PRISMA-ScR Checklist. Filled PRISMA-ScR checklist.

**Additional file 2.** Process stages of becoming an HLHCO. Synthesized process stages and examples for becoming an HLHCO.

**Additional file 3.** Involved actors by type of organization. Actors involved in the process of becoming an HLHCO by type of organization.

**Additional file 4.** Implemented interventions. Implemented interventions to promote OHL grouped by category of criteria.

**Additional file 5.** Search details. Search string used for PubMed and search settings used for CINAHL, Cochrane Library, JSTOR, PsycINFO, PubMed, Web of Science Core Collection, Wiley Online Libary.

**Additional file 6.** Included and excluded records. List of included and excluded records based on full-text screening (including records identified through snowballing).

**Additional file 7.** OHL criteria by category. Detailed list of extracted OHL criteria and records sorted by main and subcategory.

## Data Availability

The search syntax, checklist, list of extractions, and included and excluded studies are provided as additional files. Please see supplementary materials.

## Abbreviations

AHRQ: Agency for Healthcare Research and Quality
HL: Health literacy
HLHCO: Health literate health care organization
IOM: Institute of Medicine
NALA: National Adult Literacy Agency
NAM: The National Academy of Medicine (formerly IOM)
OHL: Organizational health literacy
OHL-HAM: Research project on OHL in the Hamburg Metropolitan Area (Germany)
PICo: Population, Interest, Context
PRISMA-ScR: Preferred Reporting Items for Systematic reviews and Meta-Analyses extension for Scoping Reviews Checklist

## Footnotes

1 Concepts are understood as frameworks, models or definitions of OHL which were referenced to and used within the records to derive the mentioned terminologies of OHL.
