# Supplementary material for: Which criteria characterize a health literate health care organization? – a scoping review on organizational health literacy
Source: BMC Health Serv Res. 2021 Jul 6;21:664. doi: 10.1186/s12913-021-06604-z (PMC8259028; doi:10.1186/s12913-021-06604-z)
Supplement: Supplementary file 2 — Additional file 2. Process stages of becoming an HLHCO. Synthesized process stages and examples for becoming an HLHCO. [file 12913_2021_6604_MOESM2_ESM.docx]

Process stages of becoming an HLHCO

| **Examples of activities** | **n*** |
| --- | --- |
| **1. Preparation**   - Form a team with responsible personnel [1–5], external partnerships [6], organizational coach for corporate strategy [7] - Cultivate support of leadership [2, 5, 6, 8] - Raise awareness [1–3] - Identify the focus of the review [1, 3, 4, 9, 10] | **12** |
| **2. Assessment & action plan**   - Gather information, assessment, self-rating [2, 4, 6, 7, 9–13] - Analyze and interpret results [2, 6, 12] - Prioritize [2, 9, 10] - Plan actions [2, 4, 6–8, 13]   Facilitators of assessment: commitment, time, patient's stories, engagement, strategic deployment [14] | **11** |
| **3. Action & intervention**   - Implement interventions and measures [2, 8, 11–13] - Pilot sites, roll-out [7]   Facilitators of implementation: systematic approach, longer implementation periods of up to 3 years [15] | **7** |
| **4. Evaluation & monitoring**   - Evaluate and adjust [2, 7, 11] - Monitor outcomes and audit [5, 7, 11] | **4** |
| Based on the screening of 60 records and data extraction from 22 records; *n=number of extracted items |  |

References

1. DeWalt DA, Callahan LF, Hawk VH, Broucksou KA, et al. Health Literacy Universal Precautions Toolkit. Rockville, MD; 2010.

2. Institute of Medicine. Organizational Change to Improve Health Literacy: Workshop Summary. Washington, DC; 2013.

3. Brega AG, Barnard J, Mabachi NM, Weiss BD, et al. AHRQ Health Literacy Universal Precautions Toolkit. 2nd ed. Rockville, MD; 2015.

4. Ministry of Health. Health Literacy Review: A Guide. Wellington; 2015.

5. Altin SV, Stock S. Health Literate Healthcare Organizations and their Role in Future Healthcare. Journal of Nursing & Care 2015. doi:10.4172/2167-1168.1000238.

6. Rudd RE, Anderson JE. The health literacy environment of hospitals and health centers. Boston, MA; 2006.

7. Six-Means A, Bauer TK, Teeter R, Segraves D, et al. Building a Foundation of Health Literacy with Ask Me 3™. Journal of Consumer Health on the Internet. 2012;16:180–91. doi:10.1080/15398285.2012.673461.

8. Briglia E, Perlman M, Weissman MA. Integrating health literacy into organizational structure. Physician Leadersh J. 2015;2:66–9.

9. Trezona A, Dodson S, Osborne RH. Development of the Organisational Health Literacy Responsiveness (Org-HLR) self-assessment tool and process. BMC Health Serv Res. 2018;18:N.PAG-N.PAG. doi:10.1186/s12913-018-3499-6.

10. Aaby A, Palner S, Maindal HT. Fit for Diversity: A Staff-Driven Organizational Development Process Based on the Organizational Health Literacy Responsiveness Framework. Health Lit Res Pract. 2020;4:e79-e83. doi:10.3928/24748307-20200129-01.

11. Altin SV, Lorrek K, Stock S. Development and validation of a brief screener to measure the Health Literacy Responsiveness of Primary Care Practices (HLPC). BMC Fam Pract. 2015;16:1–8. doi:10.1186/s12875-015-0336-4.

12. Jacobson KL, Gazmararian JA, Kripalani S, McMorris KJ, et al. Is Our Pharmacy Meeting Patients’ Needs? A Pharmacy Health Literacy Assessment Tool User’s Guide. Rockville, MD; 2007.

13. Weaver NL, Wray RJ, Zellin S, Gautam K, Jupka K. Advancing organizational health literacy in health care organizations serving high-needs populations: A case study. J Health Commun. 2012;17:55–66. doi:10.1080/10810730.2012.714442.

14. Brach C. The Journey to Become a Health Literate Organization: A Snapshot of Health System Improvement. Stud Health Technol Inform. 2017;240:203–37. doi:10.3233/978-1-61499-790-0-203.

15. Kaper M, Sixsmith J, Meijering L, Vervoordeldonk J, et al. Implementation and Long-Term Outcomes of Organisational Health Literacy Interventions in Ireland and The Netherlands: A Longitudinal Mixed-Methods Study. Int J Environ Res Public Health 2019. doi:10.3390/ijerph16234812.
