# Supplementary material for: Which criteria characterize a health literate health care organization? – a scoping review on organizational health literacy
Source: BMC Health Serv Res. 2021 Jul 6;21:664. doi: 10.1186/s12913-021-06604-z (PMC8259028; doi:10.1186/s12913-021-06604-z)
Supplement: Supplementary file 3 — Additional file 3. Involved actors by type of organization. Actors involved in the process of becoming an HLHCO by type of organization. [file 12913_2021_6604_MOESM3_ESM.docx]

Involved actors by type of organization

| **Involved actors within the organization by type of organization** | **Service users involved** | **Service users not involved/n. a.** | **n*** |
| --- | --- | --- | --- |
| **1. Health care systems:** Leading staff & management (e.g. Chief Medical Officer, board of directors), marketing department, task force, health care providers | Patients, family ^[1, 2]^ | ^[3–5]^ | 5 |
| **2. Health and/or social service environment:** Administrative staff, health care providers (e.g. doctors, nurses, therapists), front desk staff, leading staff, public relations, social workers | Consumers, patients, patient ombudsman/representative, family, friends, local communities, visitors ^[6–17]^ | ^[18–29]^ | 24 |
| **3. Hospitals:** Administrative staff, managers, health care providers, staff from various departments (e.g. training, health promotion, quality improvement, communication) | Clients, patients, patient ombudsman/representatives, family, visitors ^[11, 12, 14–16, 30–35]^ | ^[29, 36–39]^ | 16 |
| **4. Academic medical center**: Leading staff & management (e.g. chief executive officer, dean), researchers, librarians, task force members, departments (e.g. administration, marketing, finance, information technology), health professions (e.g. pharmacists, therapists) | Patients ^[40, 41]^ | ^[42, 43]^ | 4 |
| **5. Health centers:** Leading staff & management, task force staff, clinical support staff, departments (e.g. human resources, marketing), health care providers | Clients, patients, family, visitors ^[34, 35, 44]^ | ^[29, 45]^ | 5 |
| **6. Primary care settings:** Advanced practice registered nurses, health care professionals, physicians | Patients, parents, caregivers, general population ^[46–49]^ | ^[50, 51]^ | 6 |
| **7. Cancer care organizations:** Health professionals, patient advocates, clinicians, researchers, representatives of HCO, insurers, federal agencies | Colon and breast cancer patients ^[52]^ | ^[53]^ | 2 |
| **8. Pharmacy environments:** Staff, management, supervisors, pharmacists, technicians, administrators, clerks | Patients, customers ^[54, 55]^ | ^[56]^ | 3 |
| **9. Rehabilitation:** Staff & management |  | ^[57]^ | 1 |
| **10. Facilities for people with disabilities:** Managers & skilled staff | People with disabilities ^[58]^ |  | 1 |
| **11. Maternal and child health organizations:**  Students, educators |  | ^[59]^ | 1 |
| **12. Insurance companies**: Representatives |  | ^[4, 5]^ | 2 |
| **13. Youth work setting**: Youth workers |  | ^[60]^ | 1 |
| Based on the screening of 60 records; multiple assignments regarding the type of organization allowed *n=number of extracted records. | | | |

References

1. Brach C. The Journey to Become a Health Literate Organization: A Snapshot of Health System Improvement. Stud Health Technol Inform. 2017;240:203–37. doi:10.3233/978-1-61499-790-0-203.

2. Six-Means A, Bauer TK, Teeter R, Segraves D, et al. Building a Foundation of Health Literacy with Ask Me 3™. Journal of Consumer Health on the Internet. 2012;16:180–91. doi:10.1080/15398285.2012.673461.

3. Institute of Medicine. How Can Health Care Organizations Become More Health Literate?: Workshop Summary. Washington, DC; 2012.

4. Eigelbach B. Ten Suggested Health Literacy Attributes of a Health Care Organization. Journal of Consumer Health on the Internet. 2017;21:201–8. doi:10.1080/15398285.2017.1311606.

5. Institute of Medicine. Organizational Change to Improve Health Literacy: Workshop Summary. Washington, DC; 2013.

6. Brega AG, Hamer MK, Albright K, Brach C, et al. Organizational Health Literacy: Quality Improvement Measures with Expert Consensus. Health Lit Res Pract. 2019;3:e127-e146. doi:10.3928/24748307-20190503-01.

7. Kaphingst KA, Weaver NL, Wray RJ, Brown MLR, et al. Effects of patient health literacy, patient engagement and a system-level health literacy attribute on patient-reported outcomes: a representative statewide survey. BMC Health Serv Res. 2014;14:475. doi:10.1186/1472-6963-14-475.

8. Abrams MA, Kurtz-Rossi S, Riffenburgh A, Savage B. Building Health Literate Organizations: A Guidebook to Achieving Organizational Change. 2014. http://www.HealthLiterateOrganization.org. Accessed 15 Sep 2020.

9. Johnson A. First impressions: towards becoming a health-literate health service. Australian health review: a publication of the Australian Hospital Association. 2014;38:190–3. doi:10.1071/AH13194.

10. NALA. Literacy Audit for Healthcare Settings. Dublin: NALA; 2009.

11. Dietscher C, Pelikan JM. Health-literate hospitals and healthcare organizations-results from an Austrian Feasibility Study on the self-assessment of organizational health literacy in hospitals. In: Schaeffer D, Pelikan JM, editors. Health literacy: Forschungsstand und Perspektiven. 1st ed. Bern: Hogrefe; 2017. p. 303–314.

12. Dietscher C, Pelikan JM. Gesundheitskompetente Krankenbehandlungsorganisationen. Pravention Und Gesundheitsforderung. 2016;11:53–62. doi:10.1007/s11553-015-0523-0.

13. Thomacos N, Zazryn T. Enliven Organisational Health Literacy Self-assessment Resource. Melbourne; 2013.

14. Goldsmith JV, Wittenberg E, Parnell TA. The COMFORT Communication Model: A Nursing Resource to Advance Health Literacy in Organizations. Journal of hospice and palliative nursing: the official journal of the Hospice and Palliative Nurses Association. 2020;22:229–37. doi:10.1097/NJH.0000000000000647.

15. Pelikan JM, Dietscher C. Why should and how can hospitals improve their organizational health literacy? Bundesgesundheitsblatt Gesundheitsforschung Gesundheitsschutz. 2015;58:989–95. doi:10.1007/s00103-015-2206-6.

16. Pelikan JM, Dietscher C. Die Gesundheitskompetenz von Gesundheitseinrichtungen entwickeln: Strategien und Beispiele. Wien; 2015.

17. Ministry of Health. Health Literacy Review: A Guide. Wellington; 2015.

18. Adsul P, Wray RJ, Gautam K, Jupka K, et al. Becoming a health literate organization: Formative research results from healthcare organizations providing care for undeserved communities. Health Serv Manage Res. 2017;30:188–96. doi:10.1177/0951484817727130.

19. Baur C, Harris LM, Squire E. The U.S. National Action Plan to Improve Health Literacy: A Model for Positive Organizational Change. Stud Health Technol Inform. 2017;240:186–202. doi:10.3233/978-1-61499-790-0-186.

20. Parker RM, Hernandez LM. What makes an organization health literate? J Health Commun. 2012;17:624–7. doi:10.1080/10810730.2012.685806.

21. Pelikan JM. Health-literate health care organisations. In: Okan O, Bauer U, Levin-Zamir D, Pinheiro P, Sørensen K, editors. International handbook of health literacy: Research, practice and policy across the life-span. Bristol, United Kingdom: Policy Press; United Kingdom; 2019. p. 539–553. doi:10.5993/AJHB.31.s1.16.

22. Wong BK. Building a health literate workplace. Workplace Health Saf. 2012;60:363–9. doi:10.3928/21650799-20120726-67.

23. Altin SV, Stock S. Health Literate Healthcare Organizations and their Role in Future Healthcare. Journal of Nursing & Care 2015. doi:10.4172/2167-1168.1000238.

24. Annarumma C, Palumbo R. Contextualizing Health Literacy to Health Care Organizations. Journal of Health Management. 2016;18:611–24. doi:10.1177/0972063416666348.

25. Brach C, Keller D, Hernandez LM, Baur C, et al. Ten Attributes of Health Literate Health Care Organizations. 2012. https://nam.edu/wp-content/uploads/2015/06/BPH_Ten_HLit_Attributes.pdf. Accessed 15 Sep 2020.

26. Palumbo R, Annarumma C. The Importance of Being Health Literate: An Organizational Health Literacy Approach. Liverpool, England; 2014.

27. Trezona A, Dodson S, Osborne RH. Development of the organisational health literacy responsiveness (Org-HLR) framework in collaboration with health and social services professionals. BMC Health Serv Res. 2017;17:1–12. doi:10.1186/s12913-017-2465-z.

28. Trezona A, Dodson S, Osborne RH. Development of the Organisational Health Literacy Responsiveness (Org-HLR) self-assessment tool and process. BMC Health Serv Res. 2018;18:N.PAG-N.PAG. doi:10.1186/s12913-018-3499-6.

29. Farmanova E. Organization of health services for minority populations: The role of organizational health literacy and an active offer of health services in French in Ontario. Ottawa, Canada; 2017.

30. Kaper M, Sixsmith J, Meijering L, Vervoordeldonk J, et al. Implementation and Long-Term Outcomes of Organisational Health Literacy Interventions in Ireland and The Netherlands: A Longitudinal Mixed-Methods Study. Int J Environ Res Public Health 2019. doi:10.3390/ijerph16234812.

31. Kowalski C, Lee S-YD, Schmidt A, Wesselmann S, et al. The health literate health care organization 10 item questionnaire (HLHO-10): development and validation. BMC Health Serv Res. 2015;15:47. doi:10.1186/s12913-015-0707-5.

32. Palumbo R, Annarumma C, Musella M. Exploring the meaningfulness of healthcare organizations: a multiple case study. International Journal of Public Sector Management. 2017;30:503–18. doi:10.1108/IJPSM-10-2016-0174.

33. Hayran O, Özer O. Organizational health literacy as a determinant of patient satisfaction. Public Health. 2018;163:20–6. doi:10.1016/j.puhe.2018.06.011.

34. Rudd RE, Anderson JE. The health literacy environment of hospitals and health centers. Boston, MA; 2006.

35. Rudd RE. The Health Literacy Environment Activity Packet: First Impressions & Walking Interview. Cambridge, MA, USA; 2010.

36. Bonaccorsi G, Romiti A, Ierardi F, Innocenti M, et al. Health-Literate Healthcare Organizations and Quality of Care in Hospitals: A Cross-Sectional Study Conducted in Tuscany. Int J Environ Res Public Health 2020. doi:10.3390/ijerph17072508.

37. Innis JA. Health literate discharge practices in Ontario hospitals. US: ProQuest Information & Learning; US; 2016.

38. Napel AT. Nurses' perceptions of importance and achievability of the ten attributes of health literate healthcare organizations in their institutions: A descriptive study. 2016. https://digitalcommons.molloy.edu/etd/34. Accessed 25 Feb 2021.

39. Trueheart SL. Health literacy best practices in policy development. US: ProQuest Information & Learning; US; 2018.

40. Leonard K, Oelschlegel S, Tester E, Russomanno J, Heidel RE. Assessing the Print Communication and Technology Attributes of an Academic Medical Center. Health Lit Res Pract. 2018;2:e26-e34. doi:10.3928/24748307-20180108-01.

41. Oelschlegel S, Grabeel KL, Tester E, Heidel RE, Russomanno J. Librarians Promoting Changes in the Health Care Delivery System through Systematic Assessment. Med Ref Serv Q. 2018;37:142–52. doi:10.1080/02763869.2018.1439216.

42. Prince LY. Assessing Organizational Health Literacy at an academic health center: A quantitative research study. Fayetteville, NC: ProQuest Information & Learning; US; 2017.

43. Prince LY, Schmidtke C, Beck JK, Hadden KB. An Assessment of Organizational Health Literacy Practices at an Academic Health Center. Qual Manag Health Care. 2018;27:93–7. doi:10.1097/QMH.0000000000000162.

44. Weaver NL, Wray RJ, Zellin S, Gautam K, Jupka K. Advancing organizational health literacy in health care organizations serving high-needs populations: A case study. J Health Commun. 2012;17:55–66. doi:10.1080/10810730.2012.714442.

45. Briglia E, Perlman M, Weissman MA. Integrating health literacy into organizational structure. Physician Leadersh J. 2015;2:66–9.

46. Altin SV, Lorrek K, Stock S. Development and validation of a brief screener to measure the Health Literacy Responsiveness of Primary Care Practices (HLPC). BMC Fam Pract. 2015;16:1–8. doi:10.1186/s12875-015-0336-4.

47. DeWalt DA, Broucksou KA, Hawk VH, Brach C, et al. Developing and testing the health literacy universal precautions toolkit. Nurs Outlook. 2011;59:85–94. doi:10.1016/j.outlook.2010.12.002.

48. DeWalt DA, Callahan LF, Hawk VH, Broucksou KA, et al. Health Literacy Universal Precautions Toolkit. Rockville, MD; 2010.

49. Brega AG, Barnard J, Mabachi NM, Weiss BD, et al. AHRQ Health Literacy Universal Precautions Toolkit. 2nd ed. Rockville, MD; 2015.

50. Schuman MJ. Policy implications for advanced practice registered nurses: Quality and safety. In: Goudreau KA, Smolenski MC, editors. Health policy and advanced practice nursing: Impact and implications. New York, NY: Springer Publishing Company LLC; 2014. p. 253–271.

51. Brach C, Dreyer BP, Schillinger D. Physicians' roles in creating health literate organizations: a call to action. J Gen Intern Med. 2014;29:273–5. doi:10.1007/s11606-013-2619-6.

52. Ernstmann N, Halbach S, Kowalski C, Pfaff H, Ansmann L. Measuring attributes of health literate health care organizations from the patients' perspective: Development and validation of a questionnaire to assess health literacy-sensitive communication (HL-COM). Z Evid Fortbild Qual Gesundhwes. 2017;121:58–63. doi:10.1016/j.zefq.2016.12.008.

53. National Academies of Sciences, Engineering, and Medicine. Health Literacy and Communication Strategies in Oncology: Proceedings of a Workshop. Washington, DC; 2020 Feb 14.

54. O'Neal KS, Crosby KM, Miller MJ, Murray KA, Condren ME. Assessing health literacy practices in a community pharmacy environment: experiences using the AHRQ Pharmacy Health Literacy Assessment Tool. Research in social & administrative pharmacy: RSAP. 2013;9:564–96. doi:10.1016/j.sapharm.2012.09.005.

55. Jacobson KL, Gazmararian JA, Kripalani S, McMorris KJ, et al. Is Our Pharmacy Meeting Patients’ Needs? A Pharmacy Health Literacy Assessment Tool User’s Guide. Rockville, MD; 2007.

56. Palumbo R, Annarumma C. Empowering organizations to empower patients: An organizational health literacy approach. International Journal of Healthcare Management. 2018;11:133–42. doi:10.1080/20479700.2016.1253254.

57. Aaby A, Palner S, Maindal HT. Fit for Diversity: A Staff-Driven Organizational Development Process Based on the Organizational Health Literacy Responsiveness Framework. Health Lit Res Pract. 2020;4:e79-e83. doi:10.3928/24748307-20200129-01.

58. Rathmann K, Vockert T, Wetzel LD, Lutz J, Dadaczynski K. Organizational Health Literacy in Facilities for People with Disabilities: First Results of an Explorative Qualitative and Quantitative Study. Int J Environ Res Public Health 2020. doi:10.3390/ijerph17082886.

59. Vamos CA, Thompson EL, Griner SB, Liggett LG, Daley EM. Applying Organizational Health Literacy to Maternal and Child Health. Matern Child Health J. 2019;23:597–602. doi:10.1007/s10995-018-2687-7.

60. Wieczorek CC, Ganahl K, Dietscher C. Improving Organizational Health Literacy in Extracurricular Youth Work Settings. Health Lit Res Pract. 2017;1:e233-e238. doi:10.3928/24748307-20171101-01.
