# Supplementary material for: Which criteria characterize a health literate health care organization? – a scoping review on organizational health literacy
Source: BMC Health Serv Res. 2021 Jul 6;21:664. doi: 10.1186/s12913-021-06604-z (PMC8259028; doi:10.1186/s12913-021-06604-z)
Supplement: Supplementary file 4 — Additional file 4. Implemented interventions. Implemented interventions to promote OHL grouped by category of criteria. [file 12913_2021_6604_MOESM4_ESM.docx]

Implemented interventions to promote OHL grouped by category of criteria

| **Category and examples for intervention** | **Number of extracted interventions** |
| --- | --- |
| **1. Communication with service users**  Multilingual guidebook, patient's notebook, patient preparation sheet, easy to understand patient forms, information, registration materials, one-paged papers/materials, plain language, teach-back method, Ask Me 3, culturally and linguistically appropriate services with translation services, office/council (for diversity, inclusion, equity, health literacy) ^[1–8]^ | 25 |
| **2. Easy access & navigation**  Health literate web-content, patient outcomes and patient satisfaction results on the website, patient reminder (text messages, calls), electronic patient scheduling, navigation tracers, electronic medical record, community dental health coordinator, community outreach, and education ^[1, 3, 7, 8]^ | 8 |
| **3. Integration & prioritization of OHL**  Engagement of senior team and upper-level management, patient communications committee, health literacy task force, program manager, embedding of health literacy policies, dashboard with results of patient surveys measuring health literacy, health literacy team, patient education committee ^[5, 6, 8]^ | 9 |
| **4. Assessment & organizational development**  Patient surveys, improving medication distribution to improve patient compliance, grade providers on how well they communicate with the patient ^[4, 7, 8]^ | 4 |
| **5. Engagement & support of service users**  enhanced patient engagement, literacy volunteer program ^[6, 8]^ | 2 |
| **6. Information & qualification of staff**  Assessment concerning workforce development, surveys of staff on communication techniques, training (on communication, teach-back method, health literacy, plain language, patient safety), increasing staff and provider awareness about health literacy-related challenges experienced by patients, integrating health literacy information in orientation materials, dignity and respect campaign ^[1, 3–9]^ | 19 |
| Based on the screening of 60 records and data extraction from 9 records. | |

References

1. Baur C, Harris LM, Squire E. The U.S. National Action Plan to Improve Health Literacy: A Model for Positive Organizational Change. Stud Health Technol Inform. 2017;240:186–202. doi:10.3233/978-1-61499-790-0-186.

2. Six-Means A, Bauer TK, Teeter R, Segraves D, et al. Building a Foundation of Health Literacy with Ask Me 3™. Journal of Consumer Health on the Internet. 2012;16:180–91. doi:10.1080/15398285.2012.673461.

3. Institute of Medicine. How Can Health Care Organizations Become More Health Literate?: Workshop Summary. Washington, DC; 2012.

4. Trueheart SL. Health literacy best practices in policy development. US: ProQuest Information & Learning; US; 2018.

5. Briglia E, Perlman M, Weissman MA. Integrating health literacy into organizational structure. Physician Leadersh J. 2015;2:66–9.

6. Kaper M, Sixsmith J, Meijering L, Vervoordeldonk J, et al. Implementation and Long-Term Outcomes of Organisational Health Literacy Interventions in Ireland and The Netherlands: A Longitudinal Mixed-Methods Study. Int J Environ Res Public Health 2019. doi:10.3390/ijerph16234812.

7. Adsul P, Wray RJ, Gautam K, Jupka K, et al. Becoming a health literate organization: Formative research results from healthcare organizations providing care for undeserved communities. Health Serv Manage Res. 2017;30:188–96. doi:10.1177/0951484817727130.

8. Institute of Medicine. Organizational Change to Improve Health Literacy: Workshop Summary. Washington, DC; 2013.

9. Goldsmith JV, Wittenberg E, Parnell TA. The COMFORT Communication Model: A Nursing Resource to Advance Health Literacy in Organizations. Journal of hospice and palliative nursing: the official journal of the Hospice and Palliative Nurses Association. 2020;22:229–37. doi:10.1097/NJH.0000000000000647.
