# Supplementary material for: Which criteria characterize a health literate health care organization? – a scoping review on organizational health literacy
Source: BMC Health Serv Res. 2021 Jul 6;21:664. doi: 10.1186/s12913-021-06604-z (PMC8259028; doi:10.1186/s12913-021-06604-z)
Supplement: Supplementary file 5 — Additional file 5. Search details. Search string used for PubMed and search settings used for CINAHL, Cochrane Library, JSTOR, PsycINFO, PubMed, Web of Science Core Collection, Wiley Online Libary. [file 12913_2021_6604_MOESM5_ESM.docx]

**Search string used for PubMed**

(((organizatio*[Title/Abstract]) OR (organisatio*[Title/Abstract])) AND (("health literacy"[Title/Abstract]) OR ("health literate"[Title/Abstract]))) AND (((((((criteria[Title/Abstract]) OR (criterion[Title/Abstract])) OR (attribut*[Title/Abstract])) OR (policy[Title/Abstract])) OR (policies[Title/Abstract])) OR (guideline*[Title/Abstract])) OR (recommendation*[Title/Abstract])) AND (english[Filter] OR german[Filter])

**Search settings**

| database | records | title | abstract | language filter |
| --- | --- | --- | --- | --- |
| CINAHL | 139 | no | yes | yes |
| Cochrane Library | 1 | no | yes | no |
| JSTOR | 8 | no | yes | no |
| PsycINFO | 72 | no | yes | no |
| PubMed | 212 | yes | yes | yes |
| Web of Science Core Collection | 174 | yes | yes | no |
| Wiley Online Libary | 33 | no | yes | no |
