# Supplementary material for: Which criteria characterize a health literate health care organization? – a scoping review on organizational health literacy
Source: BMC Health Serv Res. 2021 Jul 6;21:664. doi: 10.1186/s12913-021-06604-z (PMC8259028; doi:10.1186/s12913-021-06604-z)
Supplement: Supplementary file 6 — Additional file 6. Included and excluded records. List of included and excluded records based on full-text screening (including records identified through snowballing). [file 12913_2021_6604_MOESM6_ESM.docx]

# Included records after full-text screening and snowballing process

1. Aaby A, Palner S, Maindal HT. Fit for Diversity: A Staff-Driven Organizational Development Process Based on the Organizational Health Literacy Responsiveness Framework. Health Lit Res Pract. 2020;4:e79-e83. doi:10.3928/24748307-20200129-01.

2. Abrams MA, Kurtz-Rossi S, Riffenburgh A, Savage B. Building Health Literate Organizations: A Guidebook to Achieving Organizational Change. 2014. http://www.HealthLiterateOrganization.org. Accessed 15 Sep 2020.

3. Adsul P, Wray RJ, Gautam K, Jupka K, et al. Becoming a health literate organization: Formative research results from healthcare organizations providing care for undeserved communities. Health Serv Manage Res. 2017;30:188–96. doi:10.1177/0951484817727130.

4. Altin SV, Lorrek K, Stock S. Development and validation of a brief screener to measure the Health Literacy Responsiveness of Primary Care Practices (HLPC). BMC Fam Pract. 2015;16:1–8. doi:10.1186/s12875-015-0336-4.

5. Altin SV, Stock S. Health Literate Healthcare Organizations and their Role in Future Healthcare. Journal of Nursing & Care 2015. doi:10.4172/2167-1168.1000238.

6. Annarumma C, Palumbo R. Contextualizing Health Literacy to Health Care Organizations. Journal of Health Management. 2016;18:611–24. doi:10.1177/0972063416666348.

7. Baur C, Harris LM, Squire E. The U.S. National Action Plan to Improve Health Literacy: A Model for Positive Organizational Change. Stud Health Technol Inform. 2017;240:186–202. doi:10.3233/978-1-61499-790-0-186.

8. Bonaccorsi G, Romiti A, Ierardi F, Innocenti M, et al. Health-Literate Healthcare Organizations and Quality of Care in Hospitals: A Cross-Sectional Study Conducted in Tuscany. Int J Environ Res Public Health 2020. doi:10.3390/ijerph17072508.

9. Brach C. The Journey to Become a Health Literate Organization: A Snapshot of Health System Improvement. Stud Health Technol Inform. 2017;240:203–37. doi:10.3233/978-1-61499-790-0-203.

10. Brach C, Dreyer BP, Schillinger D. Physicians' roles in creating health literate organizations: a call to action. J Gen Intern Med. 2014;29:273–5. doi:10.1007/s11606-013-2619-6.

11. Brach C, Keller D, Hernandez LM, Baur C, et al. Ten Attributes of Health Literate Health Care Organizations. 2012. https://nam.edu/wp-content/uploads/2015/06/BPH_Ten_HLit_Attributes.pdf. Accessed 15 Sep 2020.

12. Brega AG, Barnard J, Mabachi NM, Weiss BD, et al. AHRQ Health Literacy Universal Precautions Toolkit. 2nd ed. Rockville, MD; 2015.

13. Brega AG, Hamer MK, Albright K, Brach C, et al. Organizational Health Literacy: Quality Improvement Measures with Expert Consensus. Health Lit Res Pract. 2019;3:e127-e146. doi:10.3928/24748307-20190503-01.

14. Briglia E, Perlman M, Weissman MA. Integrating health literacy into organizational structure. Physician Leadersh J. 2015;2:66–9.

15. DeWalt DA, Broucksou KA, Hawk VH, Brach C, et al. Developing and testing the health literacy universal precautions toolkit. Nurs Outlook. 2011;59:85–94. doi:10.1016/j.outlook.2010.12.002.

16. DeWalt DA, Callahan LF, Hawk VH, Broucksou KA, et al. Health Literacy Universal Precautions Toolkit. Rockville, MD; 2010.

17. Dietscher C, Pelikan JM. Gesundheitskompetente Krankenbehandlungsorganisationen. Pravention Und Gesundheitsforderung. 2016;11:53–62. doi:10.1007/s11553-015-0523-0.

18. Dietscher C, Pelikan JM. Health-literate hospitals and healthcare organizations-results from an Austrian Feasibility Study on the self-assessment of organizational health literacy in hospitals. In: Schaeffer D, Pelikan JM, editors. Health literacy: Forschungsstand und Perspektiven. 1st ed. Bern: Hogrefe; 2017. p. 303–314.

19. Eigelbach B. Ten Suggested Health Literacy Attributes of a Health Care Organization. Journal of Consumer Health on the Internet. 2017;21:201–8. doi:10.1080/15398285.2017.1311606.

20. Ernstmann N, Halbach S, Kowalski C, Pfaff H, Ansmann L. Measuring attributes of health literate health care organizations from the patients' perspective: Development and validation of a questionnaire to assess health literacy-sensitive communication (HL-COM). Z Evid Fortbild Qual Gesundhwes. 2017;121:58–63. doi:10.1016/j.zefq.2016.12.008.

21. Farmanova E. Organization of health services for minority populations: The role of organizational health literacy and an active offer of health services in French in Ontario. Ottawa, Canada; 2017.

22. Goldsmith JV, Wittenberg E, Parnell TA. The COMFORT Communication Model: A Nursing Resource to Advance Health Literacy in Organizations. Journal of hospice and palliative nursing: the official journal of the Hospice and Palliative Nurses Association. 2020;22:229–37. doi:10.1097/NJH.0000000000000647.

23. Hayran O, Özer O. Organizational health literacy as a determinant of patient satisfaction. Public Health. 2018;163:20–6. doi:10.1016/j.puhe.2018.06.011.

24. Innis JA. Health literate discharge practices in Ontario hospitals. US: ProQuest Information & Learning; US; 2016.

25. Institute of Medicine. How Can Health Care Organizations Become More Health Literate?: Workshop Summary. Washington, DC; 2012.

26. Institute of Medicine. Organizational Change to Improve Health Literacy: Workshop Summary. Washington, DC; 2013.

27. Jacobson KL, Gazmararian JA, Kripalani S, McMorris KJ, et al. Is Our Pharmacy Meeting Patients’ Needs? A Pharmacy Health Literacy Assessment Tool User’s Guide. Rockville, MD; 2007.

28. Johnson A. First impressions: towards becoming a health-literate health service. Australian health review: a publication of the Australian Hospital Association. 2014;38:190–3. doi:10.1071/AH13194.

29. Kaper M, Sixsmith J, Meijering L, Vervoordeldonk J, et al. Implementation and Long-Term Outcomes of Organisational Health Literacy Interventions in Ireland and The Netherlands: A Longitudinal Mixed-Methods Study. Int J Environ Res Public Health 2019. doi:10.3390/ijerph16234812.

30. Kaphingst KA, Weaver NL, Wray RJ, Brown MLR, et al. Effects of patient health literacy, patient engagement and a system-level health literacy attribute on patient-reported outcomes: a representative statewide survey. BMC Health Serv Res. 2014;14:475. doi:10.1186/1472-6963-14-475.

31. Kowalski C, Lee S-YD, Schmidt A, Wesselmann S, et al. The health literate health care organization 10 item questionnaire (HLHO-10): development and validation. BMC Health Serv Res. 2015;15:47. doi:10.1186/s12913-015-0707-5.

32. Leonard K, Oelschlegel S, Tester E, Russomanno J, Heidel RE. Assessing the Print Communication and Technology Attributes of an Academic Medical Center. Health Lit Res Pract. 2018;2:e26-e34. doi:10.3928/24748307-20180108-01.

33. Ministry of Health. Health Literacy Review: A Guide. Wellington; 2015.

34. NALA. Literacy Audit for Healthcare Settings. Dublin: NALA; 2009.

35. Napel AT. Nurses' perceptions of importance and achievability of the ten attributes of health literate healthcare organizations in their institutions: A descriptive study. 2016. https://digitalcommons.molloy.edu/etd/34. Accessed 25 Feb 2021.

36. National Academies of Sciences, Engineering, and Medicine. Health Literacy and Communication Strategies in Oncology: Proceedings of a Workshop. Washington, DC; 2020 Feb 14.

37. Oelschlegel S, Grabeel KL, Tester E, Heidel RE, Russomanno J. Librarians Promoting Changes in the Health Care Delivery System through Systematic Assessment. Med Ref Serv Q. 2018;37:142–52. doi:10.1080/02763869.2018.1439216.

38. O'Neal KS, Crosby KM, Miller MJ, Murray KA, Condren ME. Assessing health literacy practices in a community pharmacy environment: experiences using the AHRQ Pharmacy Health Literacy Assessment Tool. Research in social & administrative pharmacy: RSAP. 2013;9:564–96. doi:10.1016/j.sapharm.2012.09.005.

39. Palumbo R, Annarumma C. The Importance of Being Health Literate: An Organizational Health Literacy Approach. Liverpool, England; 2014.

40. Palumbo R, Annarumma C. Empowering organizations to empower patients: An organizational health literacy approach. International Journal of Healthcare Management. 2018;11:133–42. doi:10.1080/20479700.2016.1253254.

41. Palumbo R, Annarumma C, Musella M. Exploring the meaningfulness of healthcare organizations: a multiple case study. International Journal of Public Sector Management. 2017;30:503–18. doi:10.1108/IJPSM-10-2016-0174.

42. Parker RM, Hernandez LM. What makes an organization health literate? J Health Commun. 2012;17:624–7. doi:10.1080/10810730.2012.685806.

43. Pelikan JM. Health-literate health care organisations. In: Okan O, Bauer U, Levin-Zamir D, Pinheiro P, Sørensen K, editors. International handbook of health literacy: Research, practice and policy across the life-span. Bristol, United Kingdom: Policy Press; United Kingdom; 2019. p. 539–553. doi:10.5993/AJHB.31.s1.16.

44. Pelikan JM, Dietscher C. Die Gesundheitskompetenz von Gesundheitseinrichtungen entwickeln: Strategien und Beispiele. Wien; 2015.

45. Pelikan JM, Dietscher C. Why should and how can hospitals improve their organizational health literacy? Bundesgesundheitsblatt Gesundheitsforschung Gesundheitsschutz. 2015;58:989–95. doi:10.1007/s00103-015-2206-6.

46. Prince LY. Assessing Organizational Health Literacy at an academic health center: A quantitative research study. Fayetteville, NC: ProQuest Information & Learning; US; 2017.

47. Prince LY, Schmidtke C, Beck JK, Hadden KB. An Assessment of Organizational Health Literacy Practices at an Academic Health Center. Qual Manag Health Care. 2018;27:93–7. doi:10.1097/QMH.0000000000000162.

48. Rathmann K, Vockert T, Wetzel LD, Lutz J, Dadaczynski K. Organizational Health Literacy in Facilities for People with Disabilities: First Results of an Explorative Qualitative and Quantitative Study. Int J Environ Res Public Health 2020. doi:10.3390/ijerph17082886.

49. Rudd RE. The Health Literacy Environment Activity Packet: First Impressions & Walking Interview. Cambridge, MA, USA; 2010.

50. Rudd RE, Anderson JE. The health literacy environment of hospitals and health centers. Boston, MA; 2006.

51. Schuman MJ. Policy implications for advanced practice registered nurses: Quality and safety. In: Goudreau KA, Smolenski MC, editors. Health policy and advanced practice nursing: Impact and implications. New York, NY: Springer Publishing Company LLC; 2014. p. 253–271.

52. Six-Means A, Bauer TK, Teeter R, Segraves D, et al. Building a Foundation of Health Literacy with Ask Me 3™. Journal of Consumer Health on the Internet. 2012;16:180–91. doi:10.1080/15398285.2012.673461.

53. Thomacos N, Zazryn T. Enliven Organisational Health Literacy Self-assessment Resource. Melbourne; 2013.

54. Trezona A, Dodson S, Osborne RH. Development of the organisational health literacy responsiveness (Org-HLR) framework in collaboration with health and social services professionals. BMC Health Serv Res. 2017;17:1–12. doi:10.1186/s12913-017-2465-z.

55. Trezona A, Dodson S, Osborne RH. Development of the Organisational Health Literacy Responsiveness (Org-HLR) self-assessment tool and process. BMC Health Serv Res. 2018;18:N.PAG-N.PAG. doi:10.1186/s12913-018-3499-6.

56. Trueheart SL. Health literacy best practices in policy development. US: ProQuest Information & Learning; US; 2018.

57. Vamos CA, Thompson EL, Griner SB, Liggett LG, Daley EM. Applying Organizational Health Literacy to Maternal and Child Health. Matern Child Health J. 2019;23:597–602. doi:10.1007/s10995-018-2687-7.

58. Weaver NL, Wray RJ, Zellin S, Gautam K, Jupka K. Advancing organizational health literacy in health care organizations serving high-needs populations: A case study. J Health Commun. 2012;17:55–66. doi:10.1080/10810730.2012.714442.

59. Wieczorek CC, Ganahl K, Dietscher C. Improving Organizational Health Literacy in Extracurricular Youth Work Settings. Health Lit Res Pract. 2017;1:e233-e238. doi:10.3928/24748307-20171101-01.

60. Wong BK. Building a health literate workplace. Workplace Health Saf. 2012;60:363–9. doi:10.3928/21650799-20120726-67.

# Excluded records after full-text screening

1. American Pharmacists Association. Medication therapy management in pharmacy practice: core elements of an MTM service model (version 2.0). Journal of the American Pharmacists Association: JAPhA. 2008;48:341–53. doi:10.1331/JAPhA.2008.08514.

2. Austvoll-Dahlgren A, Danielsen S, Opheim E, Bjørndal A, et al. Development of a complex intervention to improve health literacy skills. Health Information & Libraries Journal. 2013;30:278–93. doi:10.1111/hir.12037.

3. Badarudeen S, Sabharwal S. Assessing readability of patient education materials: current role in orthopaedics. Clin Orthop Relat Res. 2010;468:2572–80. doi:10.1007/s11999-010-1380-y.

4. Batterham RW, Buchbinder R, Beauchamp A, Dodson S, et al. The OPtimising HEalth LIterAcy (Ophelia) process: study protocol for using health literacy profiling and community engagement to create and implement health reform. BMC Public Health. 2014;14:694. doi:10.1186/1471-2458-14-694.

5. Battersby M, Lawn S, Kowanko I, Bertossa S, et al. Chronic condition self-management support for Aboriginal people: Adapting tools and training. Aust J Rural Health. 2018;26:232–7. doi:10.1111/ajr.12413.

6. Baur C. What Must Health Literacy Stakeholders Do to Build a Public Health and Population Health Agenda? Stud Health Technol Inform. 2020;269:128–41. doi:10.3233/SHTI200027.

7. Bell J, Dziekan G, Pollack C, Mahachai V. Self-Care in the Twenty First Century: A Vital Role for the Pharmacist. Adv Ther. 2016;33:1691–703. doi:10.1007/s12325-016-0395-5.

8. Bitzer EM, Dierks M-L, Heine W, Becker P, et al. Empowerment and health literacy in medical rehabilitation - recommendations for strengthening patient education. Rehabilitation (Stuttg). 2009;48:202–10. doi:10.1055/s-0029-1231060.

9. Bitzer EM, Spörhase U. Health Literacy and patient education in medical rehabilitation. Bundesgesundheitsblatt Gesundheitsforschung Gesundheitsschutz. 2015;58:983–8. doi:10.1007/s00103-015-2205-7.

10. Black S, Balatti J, Falk I. Health literacy and social capital: what role for adult literacy partnerships and pedagogy? Studies in Continuing Education. 2013;35:146–64. doi:10.1080/0158037X.2012.712038.

11. Bousquet JJ, Schünemann HJ, Togias A, Erhola M, et al. Next-generation ARIA care pathways for rhinitis and asthma: a model for multimorbid chronic diseases. Clin Transl Allergy. 2019;9:44. doi:10.1186/s13601-019-0279-2.

12. Brach C. A daughter's frustration with the dearth of patient- and family-centered care. Patient Exp J. 2014;1:43–7.

13. Brooks C, Ballinger C, Nutbeam D, Adams J. The importance of building trust and tailoring interactions when meeting older adults' health literacy needs. Disabil Rehabil. 2017;39:2428–35. doi:10.1080/09638288.2016.1231849.

14. Burke J, Lombard W, Lachance L, Kelly P, et al. Using a Gender-Responsive Approach to Generate Policy, Systems, and Environmental Changes to Improve Women's Health. World Medical & Health Policy. 2017;9:358–76. doi:10.1002/wmh3.236.

15. Burke NJ, Napoles TM, Banks PJ, Orenstein FS, et al. Survivorship care plan information needs: Perspectives of safety-net breast cancer patients. PLoS One 2016. doi:10.1371/journal.pone.0168383.

16. Chhabra R, Chisolm DJ, Bayldon B, Quadri M, et al. Evaluation of Pediatric Human Papillomavirus Vaccination Provider Counseling Written Materials: A Health Literacy Perspective. Acad Pediatr. 2018;18:S28-S25. doi:10.1016/j.acap.2017.08.004.

17. Chiriboga DA, Hernandez M. Multicultural competence in geropsychology. APA handbook of clinical geropsychology, Vol. 1: History and status of the field and perspectives on aging. 2015:379–419. doi:10.1037/14458-016.

18. Correa DJ, Milano L, Kwon C-S, Jetté N, et al. Quantitative readability analysis of websites providing information on traumatic brain injury and epilepsy: A need for clear communication. Epilepsia. 2020;61:528–38. doi:10.1111/epi.16446.

19. Dahl S, Eagle LC. Empowering or misleading? Online health information provision challenges. Marketing Intelligence & Planning. 2016;34:1000–20. doi:10.1108/MIP-07-2015-0127.

20. Debussche X. Is adherence a relevant issue in the self-management education of diabetes? A mixed narrative review. Diabetes, metabolic syndrome and obesity: targets and therapy. 2014;7:357–67. doi:10.2147/DMSO.S36369.

21. Dierks M-L, Kofahl C. The role of self-help groups and self-help organisations in promoting health literacy in the public. Bundesgesundheitsblatt Gesundheitsforschung Gesundheitsschutz. 2019;62:17–25. doi:10.1007/s00103-018-2857-1.

22. Dietscher C, Nowak P, Pelikan JM. Health Literacy in Austria: Interventions and Research. Stud Health Technol Inform. 2020;269:192–201. doi:10.3233/SHTI200032.

23. Donovan EE, Brown LE, Crook B. Patient satisfaction with medical disclosure and consent documents for treatment: Applying conceptualizations of uncertainty to examine successful attempts at communicating risk. Journal of Communication in Healthcare. 2015;8:220–32. doi:10.1179/1753807615Y.0000000008.

24. Eid M, Nahon-Serfaty I. Risk, Activism, and Empowerment: Women's Breast Cancer in Venezuela. Int J Civ Engagem Soc Change. 2015;2:43–64. doi:10.4018/IJCESC.2015010104.

25. Estacio EV, Oliver M, Downing B, Kurth J, Protheroe J. Effective Partnership in Community-Based Health Promotion: Lessons from the Health Literacy Partnership. Int J Environ Res Public Health 2017. doi:10.3390/ijerph14121550.

26. Farmanova E, Bonneville L, Bouchard L. Organizational Health Literacy: Review of Theories, Frameworks, Guides, and Implementation Issues. Inquiry: a journal of medical care organization, provision and financing. 2018;55:46958018757848. doi:10.1177/0046958018757848.

27. Farmanova E, Bouchard L, Bonneville L. Success Strategies for Linguistically Competent Healthcare: The Magic Bullets and Cautionary Tales of the Active Offer of French-Language Health Services in Ontario. Healthc Q. 2018;20:24–30. doi:10.12927/hcq.2018.25427.

28. Finlay S, Meggetto E, Robinson A, Davis C. Health literacy education for rural health professionals: shifting perspectives. Australian health review: a publication of the Australian Hospital Association. 2019;43:404–7. doi:10.1071/AH18019.

29. Fioretti BT, Reiter M, Betrán AP, Torloni MR. Googling caesarean section: a survey on the quality of the information available on the Internet. BJOG: An International Journal of Obstetrics & Gynaecology. 2015;122:731–9. doi:10.1111/1471-0528.13081.

30. Forbes A, While A, Griffiths P, Ismail K, Heller S. Organizing and delivering diabetes education and self-care support: findings of scoping project. Journal of Health Services Research & Policy. 2011;16:42–9. doi:10.1258/jhsrp.2010.010102.

31. Gill PS, Gill TS, Kamath A, Whisnant B. Readability assessment of concussion and traumatic brain injury publications by Centers for Disease Control and Prevention. Int J Gen Med. 2012;5:923–33. doi:10.2147/IJGM.S37110.

32. Gonzalez-Burboa A, Vera-Calzaretta A, Villaseca-Silva P, Muller-Ortiz H. Type 2 diabetes mellitus as a challenge for chronic disease care models in Chile. Revista Medica De Chile. 2019;147:361–6. doi:10.4067/S0034-98872019000300361.

33. Hadden KB, Hart JK, Lalla NJ, Prince LY. Systematically Addressing Hospital Patient Education. Journal of Hospital Librarianship. 2017;17:113–24. doi:10.1080/15323269.2017.1291033.

34. Hadden KB, Prince LY, Barnes CL. Health Literacy Demands of Patient-Reported Evaluation Tools in Orthopedics: A Mixed-Methods Case Study. Qual Manag Health Care. 2018;27:98–103. doi:10.1097/QMH.0000000000000165.

35. Hernandez LM, French M, Parker RM. Roundtable on Health Literacy: Issues and Impact. Stud Health Technol Inform. 2017;240:169–85. doi:10.3233/978-1-61499-790-0-169.

36. Hill SJ, Sofra TA. How could health information be improved? Recommended actions from the Victorian Consultation on Health Literacy. Australian health review: a publication of the Australian Hospital Association. 2018;42:134–9. doi:10.1071/AH16106.

37. Hill-Briggs F, Smith AS. Evaluation of diabetes and cardiovascular disease print patient education materials for use with low-health literate populations. Diabetes Care. 2008;31:667–71. doi:10.2337/dc07-1365.

38. Ho HHD, Satur J, Meldrum R. Perceptions of oral health by those living with mental illnesses in the Victorian Community ‐ The consumer's perspective. Int J Dent Hyg. 2018;16:e10-e16. doi:10.1111/idh.12278.

39. Holloway K. Health Literacy - everyone's business. Nursing Review (1173-8014). 2013;13:20.

40. Holtz BE. Evaluating the most popular diabetes websites in the USA: a content analysis. Health Promot Int 2020. doi:10.1093/heapro/daaa008.

41. Joram E, Roberts-Dobie S, Mattison S, Devlin M, et al. The Numeracy Demands of Health Education Information: An Examination of Numerical Concepts in Written Diabetes Materials. Health Commun. 2012;27:344–55. doi:10.1080/10410236.2011.586987.

42. Juarez G. Common English/Spanish Terminology Use in Radiology. Journal of Radiology Nursing. 2011;30:9–14. doi:10.1016/j.jradnu.2010.12.001.

43. Karuranga S, Sørensen K, Coleman C, Mahmud AJ. Health Literacy Competencies for European Health Care Personnel. Health Lit Res Pract. 2017;1:e247-e256. doi:10.3928/24748307-20171005-01.

44. Koh HK, Baur C, Brach C, Harris LM, Rowden JN. Toward a systems approach to health literacy research. J Health Commun. 2013;18:1–5. doi:10.1080/10810730.2013.759029.

45. Koh HK, Berwick DM, Clancy CM, Baur C, et al. New Federal Policy Initiatives To Boost Health Literacy Can Help The Nation Move Beyond The Cycle Of Costly 'Crisis Care'. Health Aff (Millwood). 2012;31:434–43. doi:10.1377/hlthaff.2011.1169.

46. Kripalani S, Wallston K, Cavanaugh KL, Osborn CY, et al. Measures to assess a health-literate organization. Washington, DC; 2014.

47. Lachance CR, Erby LAH, Ford BM, Allen VC, JR, Kaphingst KA. Informational content, literacy demands, and usability of websites offering health-related genetic tests directly to consumers. Genetics in medicine: official journal of the American College of Medical Genetics. 2010;12:304–12. doi:10.1097/GIM.0b013e3181dbd8b2.

48. Lambert K, Mullan J, Mansfield K, Koukomous A, Mesiti L. Evaluation of the quality and health literacy demand of online renal diet information. Journal of human nutrition and dietetics: the official journal of the British Dietetic Association. 2017;30:634–45. doi:10.1111/jhn.12466.

49. Lim BT, Butow P, Mills J, Miller A, et al. Challenges and perceived unmet needs of Chinese migrants affected by cancer: Focus group findings. J Psychosoc Oncol. 2019;37:383–97. doi:10.1080/07347332.2018.1551261.

50. Lloyd JE, Song HJ, Dennis SM, Dunbar N, et al. A paucity of strategies for developing health literate organisations: A systematic review. PLoS One 2018. doi:10.1371/journal.pone.0195018.

51. Loignon C, Dupéré S, Fortin M, Ramsden VR, Truchon K. Health literacy - engaging the community in the co-creation of meaningful health navigation services: a study protocol. BMC Health Serv Res. 2018;18:505. doi:10.1186/s12913-018-3315-3.

52. Ma Y, Yang AC, Duan Y, Dong M, Yeung AS. Quality and readability of online information resources on insomnia. Front Med. 2017;11:423–31. doi:10.1007/s11684-017-0524-9.

53. Mather C, Cummings E. Promoting Participatory Health: Connecting Nurses and Consumers at Point of Care to Enhance Safety and Quality in Australia. Stud Health Technol Inform. 2019;266:115–20. doi:10.3233/SHTI190782.

54. McKinney M. Emphasis on education. Higher levels of health literacy critical to better healthcare outcomes. Modern Healthcare. 2013;43:29–32.

55. Medina-Marino A, Glockner K, Grew E, Vos L de, et al. The role of trust and health literacy in nurse-delivered point-of-care STI testing for pregnant women living with HIV, Tshwane District, South Africa. BMC Public Health. 2020;20:1–9. doi:10.1186/s12889-020-08689-3.

56. Meggetto E, Kent F, Ward B, Keleher H. Factors influencing implementation of organizational health literacy: a realist review. J Health Organ Manag. 2020;34:385–407. doi:10.1108/JHOM-06-2019-0167.

57. Meggetto E, Ward B, Isaccs A. What's in a name? An overview of organisational health literacy terminology. Australian health review: a publication of the Australian Hospital Association. 2018;42:21–30. doi:10.1071/AH17077.

58. Metting E, Schrage AJ, Kocks JW, Sanderman R, van der Molen T. Assessing the Needs and Perspectives of Patients With Asthma and Chronic Obstructive Pulmonary Disease on Patient Web Portals: Focus Group Study. JMIR Form Res. 2018;2:e22. doi:10.2196/formative.8822.

59. Migliori R. Helping tomorrow's health care consumers. J Health Commun. 2013;18:3–4. doi:10.1080/10810730.2013.829139.

60. Miron-Shatz T, Mühlhauser I, Bower B, Diefenbach M, et al. Barriers to Health Information and Building Solutions. In: Gigerenzer G, Gray JAM, editors. Better Doctors, Better Patients, Better Decisions: The MIT Press; 2011. p. 191–212. doi:10.7551/mitpress/9143.003.0017.

61. Morony S, Flynn M, McCaffery KJ, Jansen J, Webster AC. Readability of Written Materials for CKD Patients: A Systematic Review. United States; 2015 Jun.

62. Nair M, Baltag V, Bose K, Boschi-Pinto C, et al. Improving the Quality of Health Care Services for Adolescents, Globally: A Standards-Driven Approach. The Journal of adolescent health: official publication of the Society for Adolescent Medicine. 2015;57:288–98. doi:10.1016/j.jadohealth.2015.05.011.

63. Neill S, Murphy K, Chapman G. Evaluating Health Literacy Environments in Australian Health Services. Asia Pacific Journal of Health Management. 2018;13:35-+. doi:10.24083/apjhm.v13i2.

64. Okan O, Bauer U, Levin-Zamir D, Pinheiro P, Sørensen K, editors. International handbook of health literacy: Research, practice and policy across the life-span. Bristol, United Kingdom: Policy Press; United Kingdom; 2019.

65. Palumbo R. Value Co-Creation and Value Co-Destruction in the Patient-Provider Relationship. The Contribution of the “Health Literacy” Perspective. 8th Annual Conference of the EuroMed Academy of Business, Innovation, Entrepreneurship and Sustainable Value Chain in a Dynamic Environment, Verona, Italy; 2015.

66. Palumbo R. Designing health-literate health care organization: A literature review. Health Serv Manage Res. 2016;29:79–87. doi:10.1177/0951484816639741.

67. Pammer CM, Wolfmayr F, Kahlert R. Case and Care Management for the Elderly. International Journal of Integrated Care (IJIC). 2018;18:1–2. doi:10.5334/ijic.s2364.

68. Pauer F, Litzkendorf S, Göbel J, Storf H, et al. Rare Diseases on the Internet: An Assessment of the Quality of Online Information. J Med Internet Res. 2017;19:e23. doi:10.2196/jmir.7056.

69. Ratzan SC, Weinberger MB, Apfel F, Kocharian G. The Digital Health Scorecard: A New Health Literacy Metric for NCD Prevention and Care. Glob Heart. 2013;8:171–9. doi:10.1016/j.gheart.2013.05.006.

70. Reid S, White C. Health literacy in New Zealand: A tale of serendipity and indigenous health. International handbook of health literacy: Research, practice and policy across the life-span. 2019:505–20.

71. Rezaei Aghdam A, Watson J, Cliff C, Miah SJ. Improving the Theoretical Understanding Toward Patient-Driven Health Care Innovation Through Online Value Cocreation: Systematic Review. J Med Internet Res. 2020;22:e16324. doi:10.2196/16324.

72. Rikard RV, Thompson MS, Head R, McNeil C, White C. Problem Posing and Cultural Tailoring: Developing an HIV/AIDS Health Literacy Toolkit With the African American Community. Health Promotion Practice. 2012;13:626–36. doi:10.1177/1524839911416649.

73. Rowlands G, Dodson S, Leung A, Levin-Zamir D. Global Health Systems and Policy Development: Implications for Health Literacy Research, Theory and Practice. Stud Health Technol Inform. 2017;240:359–91.

74. Rowlands G, Trezona A, Russell S, Lopatina M, et al. What is the evidence on the methods, frameworks and indicators used to evaluate health literacy policies, programmes and interventions at the regional, national and organizational levels? Copenhagen; 2019.

75. Ryan L, Logsdon MC, McGill S, Stikes R, et al. Evaluation of Printed Health Education Materials for Use by Low-Education Families. Journal of nursing scholarship: an official publication of Sigma Theta Tau International Honor Society of Nursing. 2014;46:218–28. doi:10.1111/jnu.12076.

76. Salem J, Paffenholz P, Bolenz C, Brandenstein M von, et al. Websites on Bladder Cancer: an Appropriate Source of Patient Information? Journal of cancer education: the official journal of the American Association for Cancer Education. 2019;34:381–7. doi:10.1007/s13187-017-1316-2.

77. Schiavo R. Health communication: From theory to practice. San Francisco, CA, US: Jossey-Bass; US; 2014.

78. Schneider N, Bäcker A, Brenk-Franz K, Keinki C, et al. Patient information, communication and competence empowerment in oncology (PIKKO) - evaluation of a supportive care intervention for overall oncological patients. Study protocol of a non-randomized controlled trial. BMC Med Res Methodol. 2020;20:1–11. doi:10.1186/s12874-020-01002-1.

79. Shohet L, Renaud L. Critical Analysis on Best Practices in Health Literacy. Can J Public Health. 2006;97:S10-S13.

80. Simmons RA, Cosgrove SC, Romney MC, Plumb JD, et al. Health Literacy: Cancer Prevention Strategies for Early Adults. Am J Prev Med. 2017;53:S73-S77. doi:10.1016/j.amepre.2017.03.016.

81. Srinivasan D. The Impact of Trust in E-Government on Electronic Health Literacy. World Medical & Health Policy. 2014;6:22–38. doi:10.1002/wmh3.86.

82. Taylor-Clarke K, Henry-Okafor Q, Murphy C, Keyes M, et al. Assessment of commonly available education materials in heart failure clinics. J Cardiovasc Nurs. 2012;27:485–94. doi:10.1097/JCN.0b013e318220720c.

83. Toscos T, Carpenter M, Flanagan M, Kunjan K, Doebbeling BN. Identifying Successful Practices to Overcome Access to Care Challenges in Community Health Centers: A "Positive Deviance" Approach. Health Serv Res Manag Epidemiol. 2018;5:2333392817743406. doi:10.1177/2333392817743406.

84. Valizadeh L, Zamanzadeh V, Ghahramanian A, Aghajari P, Foronda C. Factors influencing nurse-to-parent communication in culturally sensitive pediatric care: A qualitative study. Contemp Nurse. 2017;53:474–88. doi:10.1080/10376178.2017.1409644.

85. Vallance JK, Taylor LM, Lavallee C. Suitability and readability assessment of educational print resources related to physical activity: implications and recommendations for practice. Patient Educ Couns. 2008;72:342–9. doi:10.1016/j.pec.2008.03.010.

86. van den Broucke S, van der Zanden G, Chang P, Doyle G, et al. Enhancing the effectiveness of diabetes self-management education: the diabetes literacy project. Horm Metab Res. 2014;46:933–8. doi:10.1055/s-0034-1389952.

87. van der Heide I, Poureslami I, Mitic W, Shum J, et al. Health literacy in chronic disease management: a matter of interaction. J Clin Epidemiol. 2018;102:134–8. doi:10.1016/j.jclinepi.2018.05.010.

88. Walsh L, Hill SJ, Allan M, Balandin S, et al. A content analysis of the consumer-facing online information about My Health Record: Implications for increasing knowledge and awareness to facilitate uptake and use. Health information management: journal of the Health Information Management Association of Australia. 2018;47:106–15. doi:10.1177/1833358317712200.

89. Willis CD, Saul JE, Bitz J, Pompu K, et al. Improving organizational capacity to address health literacy in public health: a rapid realist review. Public Health. 2014;128:515–24. doi:10.1016/j.puhe.2014.01.014.

90. Xie DX, Wang RY, Chinnadurai S. Readability of online patient education materials for velopharyngeal insufficiency. Int J Pediatr Otorhinolaryngol. 2018;104:113–9. doi:10.1016/j.ijporl.2017.09.016.

91. Zanobini P, Lorini C, Baldasseroni A, Dellisanti C, Bonaccorsi G. A Scoping Review on How to Make Hospitals Health Literate Healthcare Organizations. Int J Environ Res Public Health 2020. doi:10.3390/ijerph17031036.
